# Dislocation of Three Cervical Vertebræ—Reduction and Recovery

**Published:** 1870-12

**Authors:** 


Dislocation of three Cervical Vertebræ — Reduction and Recovery.—Dr. Theodore Myers, of Belleville, Ill., reports a case in which he avows this very remarkable set of circumstances took place. A patient, a large man, was accidentally caught by his clothing upon a shaft in a mill, which revolved at the rate of sixty times per minute; he was rolled up backwards and the shaft made a good many revolutions before it could be stopped.

Two hours after the accident, Dr. Myers found the patient lying upon his back, with his head twisted to the left, the third, fourth, and fifth cervical vertebræ dislocated laterally, and at the same time so rotated upon their axis that the spinous processes occupied a nearly lateral position. There was a knoll-like projection into the curve of the neck, on the right side, above the shoulder, which proved to be the head of the humerous dislocated, forwards and inwards; the left arm and both lower extremities were almost completely paralyzed.

An assistant was instructed to make gentle traction upon the head, and the doctor, by slight pressure upon the spinous processes of the vertebræ and rotation at the same time, was enabled to effect the reduction of the displaced bones Both the patient and the surgeon "heard and felt," distinctly, the snapping of the bones as they were replaced. The head at once recovered its proper position and mobility.

Complete recovery took place in two or three weeks.—*St. Louis Medical and Surgical Journal.*

